# Decoding obesity in the brainstem

**DOI:** 10.7554/eLife.16393

**Published:** 2016-05-09

**Authors:** Jonathan Cedernaes, Joseph Bass

**Affiliations:** Division of Endocrinology, Metabolism, and Molecular Medicine, Northwestern University Feinberg School of Medicine, Chicago, United Statesjonathan.cedernaes@northwestern.edu; Division of Endocrinology, Metabolism, and Molecular Medicine, Northwestern University Feinberg School of Medicine, Chicago, United Statesj-bass@northwestern.edu

**Keywords:** feeding behavior, neuornal circuits, neuropeptides, Mouse

## Abstract

Neurons in the brainstem are the input for a neural circuit that integrates nutrient signals to control feeding behavior.

**Related research article** D'Agostino G, Lyons DJ, Cristiano C, Burke LK, Madara JC, Campbell JN, Garcia AP, Land BB, Lowell BB, Dileone RJ, Heisler LK. 2016. Appetite controlled by a cholecystokinin nucleus of the solitary tract to hypothalamus neurocircuit. *eLife* **5**:e12225. doi: 10.7554/eLife.12225

**Image** Nerve fibers (red) that originate in the lower brainstem terminate next to a subset of neurons (green) in the hypothalamus

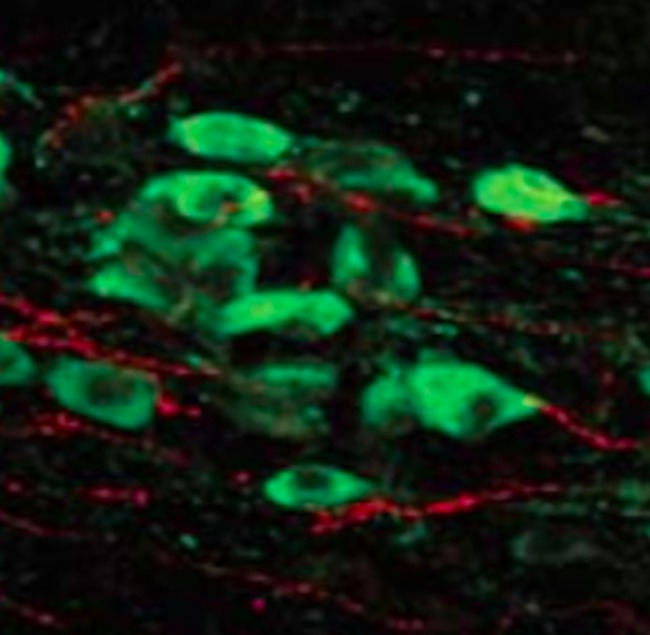

When an obese person loses weight they will often continue to experience hunger after they stop dieting and merely try to maintain a lower body weight (Sumithran et al., 2011). This situation exemplifies the complexity of regulating appetite and energy balance. Feelings of hunger or satiety result from integrating a wide range of signals from our environment with a similarly large spectrum of signals from inside the body. These internal signals might be nerve impulses from the gut, nutrient levels in the blood, or protein-based hormones. For example, fat tissue makes and releases a hormone called leptin that inhibits hunger to regulate the body’s energy balance.

The brainstem is heavily involved in these processes, integrating signals from the gut and relaying this information to a part of the brain called the hypothalamus. However, while many researchers have focused on the neurons within the feeding centers in the hypothalamus, the neurons within the brainstem have received less attention. Specifically, we don't know how the parts of the hypothalamus that respond to leptin communicate with brainstem regions that respond to more short-term signals of satiety. The specific pathways of neurons that carry and process signals from the gut have also remained less characterized.

Now, in eLife, Giuseppe D’Agostino, Lora Heisler and colleagues – who are based at the Universities of Aberdeen and Cambridge, Harvard Medical School and Yale University School of Medicine – advance this area of study by genetically dissecting a circuit of neurons that originates from the brainstem (Figure 1). D’Agostino et al. report how this circuit contributes to satiety and how it integrates with long-term control of appetite and energy balance via direct connections with neurons in the hypothalamus (D'Agostino et al., 2016).

**Figure 1.**
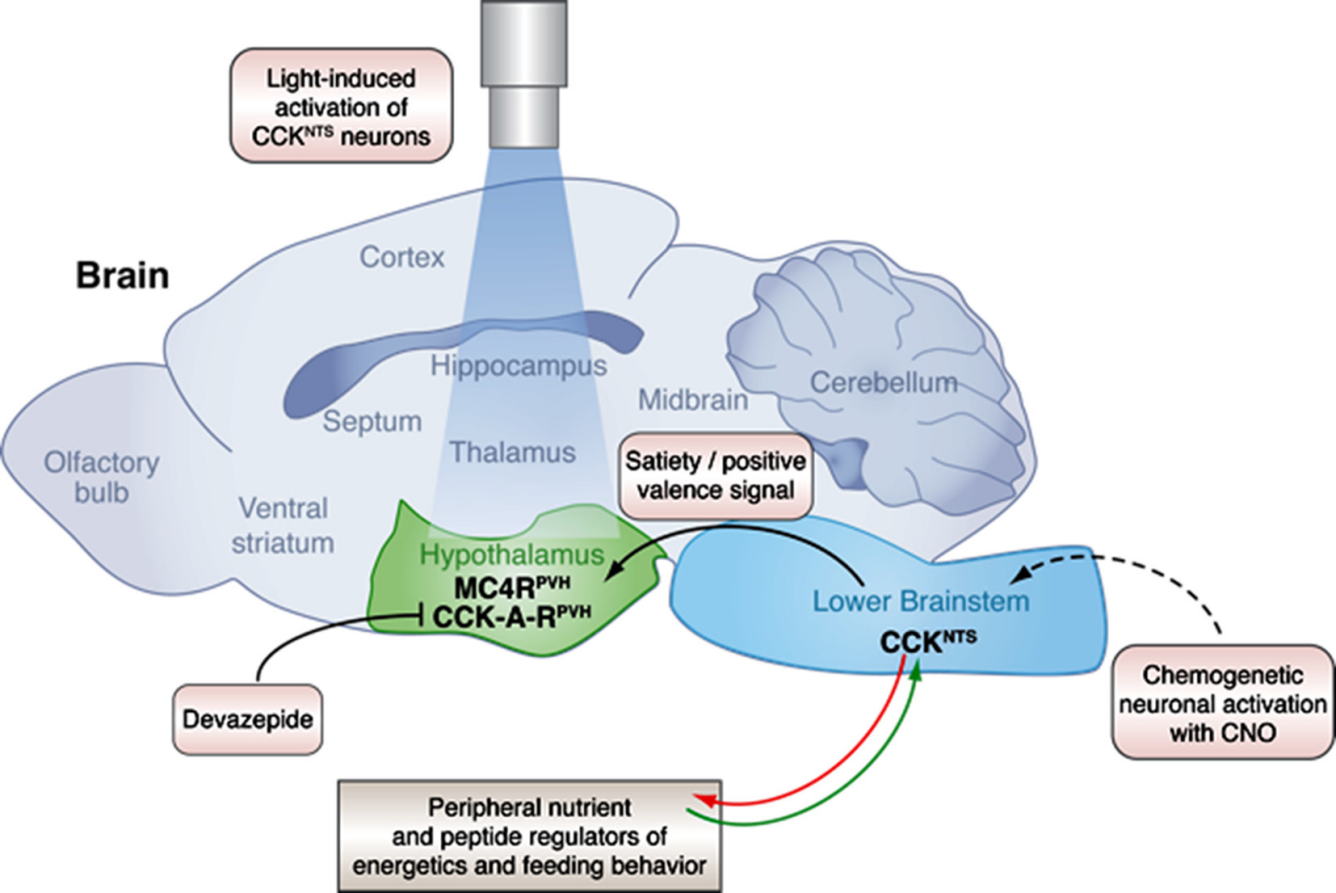
A neural circuit in the brainstem integrates signals from peripheral tissues to control feeding behavior and energy balance. The lower brainstem (light blue) receives input (green arrow) from peripheral tissues such as the gut, and also sends output (red arrow) to these tissues. Now, in experiments on mice, D’Agostino et al. have characterized a neuronal pathway that starts with neurons that express CCK in the nucleus of the solitary tract (NTS), which is part of the lower brainstem. Genetically engineering the CCK^NTS^ neurons so that a chemical called CNO can activate them showed that activating the neurons signals satiety and strongly decreases eating in mice. This leads to significant weight loss in the mice. The CCK^NTS^ neurons send signals to a part of the hypothalamus (green) called the paraventricular nucleus (PVH). Specifically, the ends of the CCK^NTS^ neurons form connections with neurons expressing melanocortin 4 receptors (MC4R^PVH^) in the PVH; the MC4R^PVH^ neurons are thought to regulate appetite. Using light to activate the endings of the CCK^NTS^ neurons that contact the PVH (via an optogenetics approach) also promoted satiety and a positive emotional state (or positive valence) in behavior tests. This strongly suggests that the connections between the CCK^NTS^ neurons and the PVH are important. Inhibiting the CCK-A receptors (CCK-A-R^PVH^) with a chemical called devazepide meant that the chemogenetic and optogenetic activation of CCK^NTS^ neurons no longer inhibited food intake. This figure was created with help from Billie Marcheva.

Over 40 years of research has implicated a molecule called cholecystokinin (or CCK) as a key regulator of satiety (Gibbs et al., 1973; Cai et al., 2014). CCK produced by the small intestine activates a part of the brainstem called the nucleus of the solitary tract (or NTS), but only if so-called melanocortin 4 receptors are expressed in this region (Fan et al., 2004). The NTS sends this nutritional information onto parts of the hypothalamus and other nearby regions of the brain (Cai et al., 2014). These brain regions then integrate signals about the animal’s nutrient state and appetite (Grill and Hayes, 2012)

D’Agostino et al. have now characterized a neural circuit in the NTS that expresses its own CCK instead. First, they showed that these CCK^NTS^ neurons became active in mice that were fed nutrients (e.g. amino acids or sucrose) but not in mice fed only water. This shows that the activity of these neurons depends on the nutrient state of the animal.

D’Agostino et al. then engineered these neurons so that they respond to a specific chemical. Activating the neurons with this chemical stopped the mice from eating even after fasting overnight. The mice lost a lot of weight if their CCK^NTS^neurons were activated for prolonged periods, but rapidly regained weight when the chemical activation was stopped. However, this chemical activator had no effect on the mice’s feeding behavior when D’Agostino et al. prevented CCK from binding to its receptors on its target neurons. This suggests that the CCK receptor is directly responsible for the signals sent from the NTS

D’Agostino et al. found that the CCK^NTS^ neurons form direct connections with the paraventricular nucleus in the hypothalamus. Stimulating this connection via an optogenetic technique stopped the mice from eating, even after they had been deprived of food. Further analysis showed the nerve endings of the CCK^NTS^ neurons at the paraventricular nucleus were also very close to a subset of paraventricular nucleus cells that express melanocortin 4 receptors.

Leptin signals how much fat is stored in the body and it was already known that this hormone regulates the activity of neurons in the brainstem that respond to CCK (Flak et al., 2014). D’Agostino et al. now give new insight into how leptin and CCK signals are integrated in the brainstem and the paraventricular nucleus, and provide further support for the idea that the connection between these signals can act in both directions (Wu et al., 2012; Morton et al., 2005; Hayes et al., 2010). Collectively, D’Agostino et al.’s findings broaden our understanding of the neuronal circuitry that processes an animal’s appetite and behavioral responses to its own energy state. The findings also describe the underlying communication, and important “gateways”, between the peripheral tissues, brainstem and hypothalamus.

At present, it is still not known what signal first activates the CCK^NTS^ neurons. This means that it remains unknown how the CCK^NTS^ neurocircuit integrates information from the gut. Also, while activating the CCK^NTS^ circuit did not promote satiety if the CCK receptors were blocked, it is not clear whether gut-derived CCK or other gut hormones can activate the CCK^NTS^ neurons to promote satiety.

The pathway characterized by D’Agostino et al. clearly has a strong effect on regulating body weight. As such, it will be important to ask whether this specific neurocircuit can be exploited to treat obesity. If this is the case, it’s worth noting that such a treatment is unlikely to use CCK itself. This is partly because this molecule is rapidly degraded in the body, and partly because people may become tolerant to CCK’s appetite-suppressing effects if it is given for prolonged periods. Importantly, other promising molecules that can activate CCK receptors have already been investigated in mice as potential weight loss therapies (Irwin et al., 2012). Perhaps these potential drugs could be improved and made more specific such that they only target a subset of CCK-expressing neurons within the gut and the brain. Thus, characterizing the CCK^NTS^ neurons further, and uncovering how they are activated by the gut, may reveal specific molecules that could be targeted by drugs to suppress appetite for long enough to combat obesity.
